# Quality of care in early detection and management of pre-eclampsia/eclampsia in health facilities in Afghanistan

**DOI:** 10.1186/s12884-018-2143-0

**Published:** 2019-01-18

**Authors:** Nasratullah Ansari, Partamin Manalai, Farzana Maruf, Sheena Currie, Jelle Stekelenburg, Jos van Roosmalen, Young-Mi Kim, Hannah Tappis

**Affiliations:** 1 Jhpiego, 1615 Thames Street, Baltimore, MD USA; 2 Athena Institute, Faculty of Science, Vrije Universiteit, Amsterdam, De Boelelaan 1105, 1081 HV Amsterdam, the Netherlands; 3 Jhpiego Afghanistan, Kabul, Afghanistan; 4 Department of Health Sciences, Global Health, University of Groningen, University Medical Centre Groningen, Groningen, the Netherlands; 5 Department of Obstetrics and Gynecology, Leeuwarden Medical Centre, Leeuwarden, the Netherlands

**Keywords:** Pre-eclampsia, eclampsia, maternal health, quality of health care, Afghanistan

## Abstract

**Background:**

Afghanistan faces a high burden of maternal and neonatal morbidity and mortality. Hypertensive disorders of pregnancy, including pre-eclampsia and eclampsia (PE/E), are among the most common causes of maternal and neonatal complications. Hypertensive disorders of pregnancy can lead to fatal complications for both the mother and fetus. The 2016 Afghanistan National Maternal and Newborn Health Quality of Care Assessment assessed quality of early detection and management of PE/E in health facilities and skilled birth attendants’ (SBAs) perceptions of their working environment.

**Methods:**

All accessible public health facilities with an average of at least five births per day (*n* = 77), a nationally representative sample of public health facilities with less than five births per day (*n* = 149), and 20 purposively selected private health facilities were assessed. Methods included a facility inventory and record review, interviews with SBAs, and direct clinical observation of antenatal care (ANC), intrapartum care and immediate postnatal care (PNC), as well as severe PE/E case management.

**Results:**

Most facilities had supplies and medicines for early detection and management of PE/E.

At public health facilities, 357 of 414 (86.2%) clients observed during ANC consultations had their blood pressure checked and 159 (38.4%) were asked if they had experienced symptoms of PE/E. Only 553 of 734 (72.6%) SBAs interviewed were able to correctly identify severe pre-eclampsia described in a case scenario. Of 29 PE/E cases observed, 17 women (59%) received the correct loading dose of magnesium sulfate (MgSO4) and 12 women (41%) received the correct maintenance dose of MgSO4.

At private health facilities, 39 of 45 ANC clients had their blood pressure checked and 9 of 45 (20%) were asked about symptoms of PE/E. Fifty-four of 64(84.4%) SBAs in private facilities correctly identified severe pre-eclampsia described in a case scenario.

**Conclusion:**

Notable gaps in SBAs’ knowledge and clinical practices in detection and management of PE/E in various health facilities increase the risk of maternal and perinatal mortality. Continuing education of health care providers and increased investment in focused quality improvement initiatives will be critical to improve the quality of health care services in Afghanistan.

## Background

Hypertensive disorders of pregnancy, including pre-eclampsia/eclampsia (PE/E), are the second most common cause of maternal mortality, accounting for 14.1% of maternal deaths, and are associated with fetal and neonatal mortality worldwide [1]. PE/E complicates 3–5% of pregnancies and occurs in the second half of pregnancy, during labor, and in the postpartum period [2]. It is one of the most common causes of preterm birth [3].

Afghanistan has one of the highest burdens of maternal mortality in the world, estimated at 789 deaths per 100,000 live births [4]. The 2010 Afghanistan Mortality Survey showed that hypertensive disorders of pregnancy account for 20% of maternal deaths, and are the second most common cause of death after obstetric hemorrhage [5]. Although the 2015 Afghanistan Demographic Health Survey did not examine causes of maternal mortality, a 2011 study showed that hypertensive disorders of pregnancy are the leading cause of maternal mortality in two districts reflective of the most urban (Kabul City) and most remote (Ragh district, Badakshan) areas in Afghanistan [6].

Administration of magnesium sulfate (MgSO4) and timely childbirth can prevent most maternal deaths due to PE/E. Ensuring timely and effective care requires appropriate use of evidence-based clinical and nonclinical interventions, strengthened health infrastructure, and motivated and competent health care providers [7, 8].

A 2010 study showed that supplies for prevention and treatment of PE/E were available in most health facilities in Afghanistan and providers were relatively well-prepared to manage severe PE/E cases [9]. Since 2010, interventions to improve quality and coverage of maternal and newborn health (MNH) care have been implemented, but the security situation in Afghanistan has deteriorated [10], resulting in facility closure and increased staff turnover [11]. Impacts on facility readiness and quality detection and management of PE/E at different levels of public health facilities are not known, and no study to date has examined readiness or quality of detection and management of PE/E at private health facilities.

This study examines the quality of early detection and management of PE/E in public and private health facilities in 2016, documents SBAs’ perceptions of their working environment, and assesses whether quality of care varies by different levels of public health facilities. Such information is crucial to identify strengths and weaknesses in the quality of care in public and private health facilities, to help to frame policy discussions, and to design evidence-based interventions to address these challenges in the country.

## Methods

### Study design

The 2016 Afghanistan Maternal and Newborn Health Quality of Care Assessment was a cross-sectional assessment examining facility readiness and quality of routine MNH services and management of obstetric and newborn complications. This study uses the subset of data on early detection and management of PE/E.

The 2016 Afghanistan Maternal and Newborn Health Quality of Care Assessment included three data collection methods: a facility inventory and record-review tool to verify availability of medications, supplies, and equipment, as well as human resources, infrastructure, and facility records; an interview tool to collect information on SBAs’ knowledge, practices, and attitudes, and constraints faced with the provision of antenatal care (ANC), labor/birth, and postnatal care (PNC) services; and direct clinical observation checklists to document ANC, labor/birth, inpatient PNC before discharge, and management of selected obstetric and newborn complications, including severe PE/E.

Observation checklist content was based on World Health Organization (WHO) guidelines and adapted from tools used in conducting quality of care assessments in other countries [12]; the content of other tools was adapted from Demographic and Health Survey Service Provision Assessment [13], and emergency obstetric and newborn care (EmONC) assessments supported by the Averting Maternal Death and Disability (AMDD) program [14]. All tools were developed in English and translated into Dari and Pashto.

### Sample

Ministry of Public Health (MoPH) guidelines require specialized hospitals (SHs), regional hospitals (RHs) provincial hospitals (PHs) and district hospitals (DHs) to provide all comprehensive EmONC signal functions [15]; and primary health care facilities including as comprehensive health centers (CHCs), basic health centers (BHCs), sub-health centers (SHCs) and family health houses [FHH] to provide all basic EmONC signal functions, including early detection and management of PE/E and refer women with complications to higher levels of care when needed [16, 17].

**Fig. 1:**
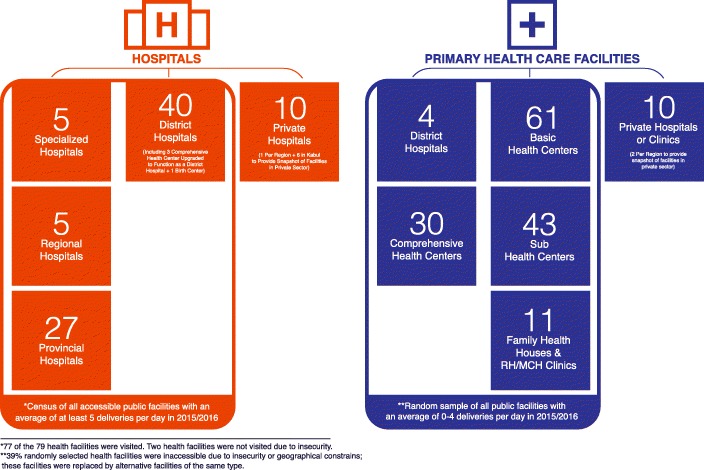
Health Facility Sample

The 2016 Afghanistan Maternal and Newborn Health Quality of Care Assessment was designed to assess readiness and quality of care at a census of all public facilities with an average of five or more deliveries per day, and to assess readiness at a nationally representative sample of public facilities with an average of zero to four deliveries per day (Fig. 1). Facility caseloads were determined based on data reported in the national health management information system: 79 public health facilities reported an average of at least five deliveries per day, 386 reported an average of 1–4 deliveries per day, and 1351 did not report any deliveries in the year prior to the assessment.

To obtain a nationally representative sample of public facilities with fewer than five births per day, we used probability proportional to size methods of cluster sampling to estimate results by facility type. Using a design effect of 1.5%, we estimated that a total of 147 public health facilities would need to be assessed to estimate results with + 10% precision, 95% confidence: four DHs, 30 CHCs, 61 BHCs, 43 SHCs, and 11 FHHs.

Twenty private sector facilities (10 hospitals with at least five deliveries per day, and 10 health centers with fewer than five deliveries per day) were purposively sampled to provide a snapshot of private facilities in all regions of the country. This sample is not statistically representative for all private sector facilities. The private health sector sampling is not comparable to public health sector sampling.

### Data collection, sites, and procedure

Data collectors were 32 experienced female SBAs (midwives and doctors) who successfully completed a May 2016 training on conducting clinical observations. Training sessions, which were held in Kabul, consisted of technical updates on maternal and newborn care (routine and selected complications), clinical site data collection techniques with a focus on clinical observation as well as data quality assurance, research ethics, and using CommCare software for data collection.

Data collection at health facilities with an average of at least five deliveries per day (including facility inventory/record review, SBA interviews, and direct observations of clinical care) was completed between May 14, 2016 and August 3, 2016. Data collection at health facilities that averaged fewer than five deliveries per day (facility inventory/record review and SBA interviews only) was completed from November 5, 2016 to January 5, 2017.

Data collection teams visited 77 of the 79 public health facilities with an average of at least five deliveries per day. Data collectors were not able to visit two health facilities due to insecurity surrounding them. Each team consisted of three data collectors and aimed to complete data collection within two to three days per facility. Due to insecurity or geographical and climatic constraints, 39% of randomly selected public health facilities with zero to four deliveries per day were inaccessible; therefore, these facilities were replaced by alternative facilities of the same type, following standardized replacement sampling protocols.

Interviews were conducted with 333 health providers at SHs, RHs, and PHs; 228 at high volume DHs with more than five deliveries per day; 69 at low volume DHs and CHCs; 104 at BHCs, SHCs, and FHHs; and 64 at private health facilities. Four hundred and fourteen ANC observations were conducted at public health facilities and 45 were completed at private health facilities; 507 childbirth observations took place at public health facilities and 31 at private health facilities; and 402 PNC observations were conducted at public health facilities and 30 at private health facilities.

Data collection teams in both phases used CommCare software on Android tablet computers. Where use of the tablets was considered a security risk (for example, where there was a risk of robbery), data collectors used paper tools, but loaded the contents of these tools to the tablets when they were in a safe location with Internet access. Logic, skip, and consistency checks were built into the program, and data collectors were trained to review records for missing or inconsistent answers before submission.

### Data analysis

For analysis purposes, facility types were defined as follows: 1) specialized, regional, and provincial hospitals; 2) district hospitals with an average of five or more deliveries per day; 3) district hospitals and comprehensive health centers with an average of less than five deliveries per day; and 4) basic health centers, sub-health centers, family health houses, and other primary health care facilities. These categories ensure that indicators based on direct observation of clinical services can be estimated with + 10% precision, and that facilities with similar management mechanisms and service packages are grouped together.

Descriptive statistics were used for analysis. A chi-squared test was used to test for differences in facility readiness and routine care practices by public facility type. All statistical analyses were conducted using Stata®. Data from private facilities are based on purposive nonrandom sampling and are not intended for direct comparison compared with the public health facility random sampling data.

PE/E case management data analysis focused on four key areas in only public health facilities: blood pressure (BP) and proteinuria testing and anticonvulsant and antihypertensive therapy. In both severe pre-eclampsia and eclampsia, BP check, proteinuria tests and administration of antihypertensive data was analyzed. For anticonvulsant therapy, correct loading and maintenance doses of MgSO4 were assessed based on national case management guidelines aligned with WHO Managing Complications in Pregnancy and Childbirth (MCPC) 2007 guidelines [18].

### Ethical considerations

The ethical review boards of the Afghanistan MoPH and John Hopkins Bloomberg School of Public Health (JHSPH) in Baltimore, Maryland approved the 2016 National Maternal and Newborn Health Quality of Care Assessment protocol (MoPH IRB #36153, JHSPH IRB #6799). Written permission for data collection was obtained from facility directors, and oral informed consent was obtained from all participating health care providers and clients (or a client’s next of kin if the woman was too ill to provide informed consent directly).

## Results

### Characteristics of health facilities and health care providers

In 144 of 226 public health facilities (63.7%), facility management reported providing 24-h services, but availability of 24-h care was higher (100%) in SH/RH/PHs and high volume DHs, and lower (56.8%) in low volume DH/CHC and (41.2%) in BHC/SHC/FHH (*p* < 0.001). Of 734 SBAs interviewed, 173 (23.6%) had received training in BEmONC in the last 3 years, and 186 (25.3%) had received training specifically in management of PE/E. (Table 1).

**Table 1:** Characteristics of health facilities and health care providers

Characteristics of health facilities	Facility type						
	SH^a^, RH^b^, PH^c^ (*n* = 37)	High Volume DH^d^ (5 or more deliveries per day) (*n* = 40)	Low Volume DH^d^ and CHC^e^ with 0–4 deliveries per day (*n* = 37)	BHC^f^, SHC^g^, FHH^h^ (*n* = 112)	*p*-value	All public sector (*n* = 226)	Private hospitals (*n* = 20)
# new antenatal care clients per month (median [range)	193 (0–2387)	153 (0–771)	28 (0–101)	49 (1–238)	< 0.001	56 (0–2387)	25 (0–101)
# facility births per month (median [range)	558 (76–2157)	232 (142–1233)	6 (0–33)	9 (1–98)	< 0.001	17 (0–2157)	49 (4–218)
# % facilities that provide 24-h coverage for delivery of services	100.0 (37)	100.0 (40)	56.8 (21)	41.2 (46)	< 0.001	63.7 (144)	90.0 (18)
# hours that women generally stay at health facilities following a normal delivery (average [SD]^i^)	5.0 (3.9)	5.5 (4.8)	4.8 (1.8)	4.6 (2.9)	0.596	4.9 (3.4)	5.5 (4.7)
Characteristics of health care providers number (%)	SH^a^, RH^b^, PH^c^ (*n* = 333)	DH^d^ with 5 or more deliveries per day (*n* = 228)	DH^d^ and CHC^e^ with 0–4 deliveries per day (*n* = 69)	BHC^f^, SHC^g^, FHH^h^ (*n* = 104)	*p*-value	All public sector (*n* = 734)	Private hospitals (*n* = 64)
Skilled birth attendants (SBAs) who provide antenatal services in their current positions	291 (87.4%)	198 (86.8%)	68 (98.6%)	102 (98.1%)	< 0.001	659 (89.8%)	58 (90.6%)
SBAs that provide intrapartum services in their current positions	310 (93.1%)	204 (89.5%)	67 (97.1%)	96 (92.3%)	0.007	677 (92.2%)	62 (96.9%)
SBAs that received training in the last 3 years on the following topics:							
Antenatal care screening (e.g., checking blood pressure and testing proteinuria)	91 (27.3%)	56 (24.6%)	22 (31.9%)	36 (34.6%)	0.181	205 (27.9%)	18(28.1%)
Management of pre-eclampsia/eclampsia	98 (29.4%)	42 (18.4%)	18 (26.1%)	28 (26.9%)	0.003	186 (25.3%)	16 (25.0%)
Basic emergency obstetric and newborn care	90 (27.0%)	42 (18.4%)	15 (21.7%)	26 (25.0%)	0.129	173 (23.6%)	16 (25.0%)

^a^Specialty Hospital; ^b^Regional Hospital; ^c^Provincial Hospital; ^d^District Hospital; ^e^Comprehensive Health Center; ^f^Basic Health Center; ^g^Sub-Health Center; ^h^Family Health House; ^i^standard deviation

### Availability of supplies, equipment, and drugs

Of 226 public health facilities, 178 (78.8%) had injectable MgSO4. The availability of MgSO4 did not differ significantly between facility types (*p* = 0.370). Injectable calcium gluconate, the antidote for MgSO4, was available in 113 of 226 (50.0%) health facilities, including more than 80% of SHs/RHs/PHs and high volume DHs, and less than 35% low volume DHs/ CHCs and BHCs/SHCs/FHHs (*p* < 0.001) (Table 2).

**Table 2:** Supply, equipment and drug for early detection and management of pre-eclampsia/eclampsia (PE/E) available at the point of care

Available items number (%)	Facility type						
	SH^a^, RH^b^, PH^c^ (*n* = 37)	High Volume DH^d^ (5 or more deliveries per day) (*n* = 40)	Low Volume DH^d^ and CHC^e^ (0–4 deliveries per day) (*n* = 37)	BHC^f^, SHC^g^, ^h^FHH (*n* = 112)	*p*-value	All public sector (*n* = 226)	Private hospitals (*n* = 20)
Guidelines or national treatment protocol for emergency obstetric and newborn care	16 (43.2%)	19 (47.5%)	20 (54.1%)	47 (42.0%)	0.527	102 (45.1%)	7 (35.0%)
Functioning blood pressure apparatus^i^	30 (81.1%)	34 (85.0%)	35 (94.6%)	95 (84.8%)	0.303	194 (85.8%)	15 (75.0%)
Functioning stethoscope	29 (78.4%)	36 (90.0%)	35 (94.6%)	99 (88.4%)	0.702	199 (88.1%)	17(85.0%)
Functioning fetal stethoscope or fetoscope	25 (67.6%)	36 (90.0%)	34 (91.9%)	99 (88.4%)	0.094	194 (85.8%)	15 (75.0%)
Injectable oxytocin	33 (89.2%)	36 (90.0%)	36 (97.3%)	94 (83.9%)	0.637	199 (88.1%)	18 (90.0%)
Misoprostol	21 (56.8%)	21 (52.5%)	5 (13.5%)	10 (8.9%)	< 0.001	57 (25.2%)	12 (60.0%)
Injectable diazepam	28 (75.7%)	29 (72.5%)	25 (67.6%)	49 (43.8%)	< 0.001	131 (58.0%)	11 (55.0%)
Injectable magnesium sulfate	33 (89.2%)	36 (90.0%)	28 (75.7%)	81 (72.3%)	0.370	178 (78.8%)	18 (90.0%)
Injectable calcium gluconate	31 (83.8%)	32 (80.0%)	11 (29.7%)	39 (34.82%)	< 0.001	113 (50.0%)	15 (75.0%)

^a^Specialty Hospital; ^b^Regional Hospital; ^c^Provincial Hospital; ^d^District Hospital; ^e^Comprehensive Health Center; ^f^Basic Health Center; ^g^Sub-Health Center; ^h^Family Health House; ^i^blood pressure apparatus includes any type of sphyngomanometer

The majority of public health facilities assessed had injectable oxytocin in the delivery room (199 of 226; 88.1%); misoprostol was available in over 50% of PH/RH/SH and high volume DH, and less than 14% of low volume DH/CHC and BHC/SHC/FHH (*p* < 0.001) (Table 2).

Of 226 public health facilities, 194 (85.8%) had functioning BP machines, 199 (88.1%) had functioning stethoscopes, and 194 (85.8%) had functioning fetoscopes. (Table 2). Less than half of the health facilities (102 of 226; 45.1%) had EmONC guidelines and protocols available in the delivery rooms (Table 2).

### SBA knowledge on early detection and management of PE/E

SBAs were given descriptions of cases with obstetric complications and asked to provide a diagnosis. Overall, 553 of 734 (72.6%) SBAs correctly identified a case with severe headache, blurred vision, BP 160/120, and 3+ proteinuria as severe pre-eclampsia (Table 3).

**Table 3:** Skilled birth attendant (SBA) knowledge on early detection and management of pre-eclampsia/eclampsia (PE/E)

SBA demonstrating knowledge of PE/E early detection and management number (%)	Facility type						
	SH^a^, RH^b^, PH^c^ (*n* = 333)	High Volume DH^d^ (5 or more deliveries per day) (*n* = 228)	Low Volume DH^d^ and CHC^e^ (0–4 deliveries per day (*n* = 69)	BHC^f^, SHC^g^, FHH^h^ (*n* = 104)	*p*-value	All public sector *n* = 734)	Private hospitals (*n* = 64)
Know essential actions to take in management of severe pre-eclampsia at term							
Administer magnesium sulfate	272 (81.7%)	176 (77.2%)	66 (95.7%)	92 (88.5%)	< 0.001	606 (82.6%)	56 (87.5%)
Administer antihypertensives	205 (61.6%)	132 (57.9%)	33 (47.8%)	47 (45.2%)	< 0.001	417 (56.8%)	34 (53.1%)
Prepare to deliver within 24 h	167 (50.2%)	106 (46.5%)	14 (20.3%)	31 (29.8%)	< 0.001	318 (43.3%)	27 (42.2%)
Incorrectly responded and listed administering diazepam as appropriate management of severe pre-eclampsia at term	99 (29.7%)	63 (27.6%)	44 (63.8%)	56 (53.9%)	< 0.001	262 (35.7%)	25 (39.1%)
Know to check maternal blood pressure during postnatal care	241 (72.4%)	170 (74.6%)	61 (88.4%)	90 (86.5%)	< 0.001	562 (76.6%)	52 (81.3%)
Know correct diagnosis of severe pre-eclampsia from a case description	224 (67.3%)	158 (69.3%)	60 (87.0%)	91 (87.5%)	0.001	553 (72.6%)	54 (84.4%)
Know the following procedures that are carried out routinely for all patients during labor and delivery							
Know to monitor maternal blood pressure	258 (77.5%)	170 (74.6%)	66 (95.7%)	92 (88.5%)	< 0.001	586 (79.8%)	56 (87.5%)
Know to monitor intermittent fetal heart rate (approx. every hour or more often)	236 (70.9%)	170 (74.6%)	65 (94.2%)	90 (86.5%)	< 0.001	561 (76.4%)	52 (81.3%)

^a^Specialty Hospital; ^b^Regional Hospital; ^c^Provincial Hospital; ^d^District Hospital; ^e^Comprehensive Health Center; ^f^Basic Health Center; ^g^Sub-Health Center; ^h^Family Health House;

Of 734 SBAs interviewed, 606 (82.6%) knew that MgSO4 was essential for management of severe pre-eclampsia. SBAs’ knowledge of MgSO4 varied significantly by health facility type, ranging from 272 of 333 (81.7%) in PH/RH/SH and 176 of 228 (77.2%) high volume DHs to 66 of 69 (95.7%) in low volume DH/CHC and 92 of 104 (88.5%) in BHC/SHC/FHH (*p* = 0.001). In addition, 318 of 734 (43.3%) SBAs knew that preparing women for childbirth within 24 h was an essential action in the management of severe pre-eclampsia, with over 56% of SBAs from PH/RH/SH and high volume DH and less than 30% of SBAs from low volume DH/CHC and BHC/SHC/FHH demonstrating this knowledge (*p* < 0.001). Moreover, 417 of 734 (56.8%) SBAs knew that antihypertensive medication was essential for management of severe pre-eclampsia, with more than 57% of SBAs from PH/RH/SH and high volume DH and less than 48% of SBAs from low volume DH/CHC and BHC/SHC/FHH answering questions about this correctly (*p* < 0.001) (Table 3).

### Direct observation during ANC, labor/birth, and inpatient PNC for early detection of PE/E

SBAs in public health facilities measured BP of 357 of 414 (86.2%) women observed during ANC, 320 of 507 (63.1%) women observed during the first and second stage of labor, and 290 of 402 (72.1%) women observed in the postnatal ward.

SBAs asked if women had recently or were currently experiencing severe headache or blurred vision in 159 of 414 (38.4%) ANC consultations observed, 59 of 436 (13.5%) women observed during childbirth and 124 of 402 (30.8%) women observed in the postnatal ward.

SBAs counseled women on the need for care seeking if experiencing convulsions and loss of consciousness in 108 of 414 (26.1%) ANC consultations and 115 of 402 (28.6%) inpatient PNC consultations before discharge after childbirth. Similarly, they provided counseling on the need for care seeking if experiencing severe headache with blurred vision in 150 of 414 (36.2%) of ANC consultations and 143 of 402 (35.6%) of PNC consultations (Table 4).

**Table 4:** Early detection of pre-eclampsia/eclampsia (PE/E) during antenatal care (ANC), childbirth and postnatal care (PNC) observed on assessment day

Cases with key PE/E practices observed number # (%)	Facility type					
	Antenatal care		Childbirth		Childbirth	
	Public	Private	Public	Private	Public	Private
Blood pressure measurement	357/414 (86.2%)	39/45 (86.7%)	320/507 (63.1%)	27/31 (87.1%)	290/402 (72.1%)	23/30 (76.7%)
Fetal heart rate check (at least once)	–	–	299/507 (59.0%)	25/31 (80.7%)	N/A	N/A
Assessment for danger signs						
Severe headache/blurred vision	159/414 (38.4%)	9/45 (20.0%)	59/436 (13.5%)	7/24 (29.2%)	124/402 (30.8%)	5/30 (16.7%)
Convulsion/loss consciousness	87/414 (21.0%)	1/45 (2.2)	24/436 (5.5%)	1/24 (4.2%)	80/402 (19.9%)	5/30 (16.7%)
Difficulty in breathing	117/414 (28.3%)	2/ 45 (4.4%)	43/436 (9.9%)	0/24 (0.0%)	72/402 (17.9%)	3/30 (10.0%)
Counseling on PE/E danger signs and care seeking						
Convulsions or loss of consciousness	108/414 (26.1%)	3/45 (6.7%)	–	–	115/402 (28.6)	6/30 (20%)
Severe headache with blurred vision	150/414 (36.2%)	3/45 (6.7%)	–	–	143/402 (35.6%)	6/30 (20%)
Fast or difficult breathing	107/414 (25.9%)	0/45 (0.0%)	–	–	76/402 (18.9%)	5/30 (16.7%)
When to return for next visit	278/414 (67.2%)	22/45 (48.9%)	–	–	–	–
Importance of at least having four ANC visits	208/414 (50.2%)	10/45 (22.4%)	N/A	N/A	N/A	N/A

### Direct observation of severe PE/E case management

Data collectors documented management of 33 cases of PE/E during visits to public health facilities with an average of at least five births per day. These 33 observations took place at 22 different health facilities; 26 were observed at SH/RH/PHs and seven at high volume DHs. Two women’s conditions were classified as mild pre-eclampsia and another two cases had insufficient data for classification. These four women were excluded from analysis. Of the 29 remaining PE/E cases, 18 women’s conditions were identified as severe pre-eclampsia and 11 as eclampsia (Table 5).

**Table 5:** Pre-eclampsia/eclampsia case management observed on assessment day

Number	Severe pre-eclampsia *n* = 18	Eclampsia *n* = 11	Total *n* = 29
Checking blood pressure (BP)			
Blood pressure checked	18	10	28
Unknown	0	1	1
Checking proteinuria			
Proteinuria checked	17	9	26
Unknown	1	2	3
Anticonvulsant therapy			
Administration of magnesium sulfate (MgSO4) loading dose			
MgSO4 loading dose administered *correctly*	10	7	17
MgSO4 loading dose administered *incorrectly*	5	3	8
Received a loading dose MgSO4 *without indication*	–	–	–
Insufficient data to determine if loading dose was administered correctly	3	1	4
Administration of magnesium sulfate (MgSO4) maintenance dose			
MgSO4 maintenance dose administered *correctly*	7	5	12
MgSO4 maintenance dose administered *incorrectly*	7	4	11
Insufficient data to determine if maintenance dose was administered correctly	4	2	6
Antihypertensive therapy			
Administration of antihypertensive drugs			
Antihypertensive drug(s) *administered*	13	7	20
Antihypertensive drug(s) *not administered*	5	3	8
Unknown	0	1	1

Twenty-eight of 29 women with severe PE/E had their BP measured. In one woman, the assessor did not observe BP measurement and could not ascertain from the woman’s records whether her BP was checked.

Of 29 women with severe PE/E, 17 (59%) received the correct MgSO4 loading dose (Table 5). Four women had insufficient data recorded to determine if the correct dose was administered. Of 29 women with severe PE/E, 12 women (41%) received the correct maintenance dose of MgSO4. Six cases had insufficient data recorded to determine correct maintenance dose.

The PE/E case management observation checklist documented whether antihypertensives were administered but did not collect information on dosage. Of 29 women with severe PE/E, 20 (69%) received and eight (28%) did not receive any type of antihypertensive drug. In one case of eclampsia, it is unknown if the woman received antihypertensives (Table 5).

### SBA perceptions of working conditions

Of 734 SBAs interviewed at public health facilities, 497 (67.7%) reported feeling that their supervisors treat them with respect. More than half of SBAs interviewed (434 of 734; 59.1%) mentioned the need for more knowledge, including approximately 60% of SBAs at PH/RH/SH and high volume DH and over 72% of respondents from low volume DH/CHC and BHC/SHC/FHH (*p* < 0.001). One-third of respondents (249 of 734; 33.9%) mentioned good security in the workplace as an important factor in ensuring an enabling environment for quality care provision, including more than 42% of SBAs from high volume DHs and almost 34% of SBAs from all other facility types indicated this need (*p* = 0.009) (Table 6).

**Table 6:** Skilled birth attendants’ (SBA) perception on enabling environment

SBAs’ perception on factor for improving their work environment number (%)	SH^a^/RH^b^/PH^c^ (*n* = 333)	High Volume DH^d^ (5 or more deliveries per day) (*n* = 228)	Low Volume DH^d^ and CHC^e^ (0–4 deliveries per day) (*n* = 69)	BHC^f^, SHC^g^, FHH^h^ (*n* = 104)	*p*-value	All public sector (*n* = 734)	Private hospitals (*n* = 64)
SBAs who thought that their supervisors treat them respectfully	218 (65.5%)	144 (63.2%)	51 (73.9%)	84 (80.8%)	0.014	497 (67.7%)	45 (70.3%)
SBAs identifying the most important factors for improving their work environment to provide good quality health care services							
More knowledge/updates/training	197 (59.2%)	108 (47.4%)	50 (72.5%)	79 (76.0%)	< 0.001	434 (59.1%)	47 (73.4%)
Good quality equipment/supplies	94 (28.2%)	68 (29.8%)	32 (46.4%)	33 (31.7%)	0.158	227 (30.9%)	11 (17.2%)
Good security situation	100 (30.0%)	97 (42.5%)	23 (33.3%)	29 (27.9%)	0.009	249 (33.9%)	10 (15.6%)
More support from supervisor	100 (30.0%)	84 (36.8%)	23 (33.3%)	34 (32.7%)	0.414	241 (32.8%)	18 (28.1%)
SBAs noting aspects of life outside work that would affect their ability to perform their jobs							
Childcare responsibilities	79 (23.7%)	43 (18.9%)	8 (11.6%)	17 (16.4%)	0.073	147 (20.0%)	2 (3.1%)
Household responsibilities	78 (23.4%)	56 (24.6%)	6 (8.7%)	10 (9.6%)	0.001	150 (20.4%)	5 (7.8%)
Safety in getting to/from work	63 (18.9%)	60 (26.3%)	16 (23.2%)	29 (27.9%)	0.113	168 (22.9%)	9 (14.1%)
Lack of transport	59 (17.7%)	45 (19.7%)	17 (24.6%)	31 (29.8%)	0.049	152 (20.7%)	10 (15.6%)

^a^Specialty Hospital; ^b^Regional Hospital; ^c^Provincial Hospital; ^d^District Hospital; ^e^Comprehensive Health Center; ^f^Basic Health Center; ^g^Sub-Health Center; ^h^Family Health House

Almost a quarter of SBAs (168 of 734; 22.9%) expressed concerns about safety while traveling to public health facilities. Lack of transport was another challenge emphasized by 152 of 734 (20.7%), including almost 20% of SBAs working in PH/RH/SH and high volume DH, and over 24% of respondents from low volume DH/CHC and BHC/SHC/FHH (*p* = 0.049).

### Snapshot of private health facilities

In 18 of 20 (90%) private health facilities, facility management reported providing 24-h services. Sixteen of 64 SBAs (25%) had received training in both BEmONC and PE/E management in the last 3 years (Table 1).

Of the 20 private health facilities, 18 (90%) had injectable MgSO4, 15 (75%) had injectable calcium gluconate, 18 (90%) had oxytocin and 12 (60%) had misoprostol available in the delivery room. Fifteen (75%) had functioning BP apparatus, 17 of 20 (85%) had a functioning stethoscope, 15 (75%) had a functioning fetoscope and 7 (35%) had EmONC guidelines and protocols in the delivery room (Table 2).

Of 64 SBAs interviewed at private facilities, 54 (84.4%) correctly identified a case with severe headache, blurred vision, BP 160/120, and 3+ proteinuria as severe pre-eclampsia. In addition, 56 of 64 (87.5%) SBAs recognized administration of MgSO4 as an essential action for management of severe pre-eclampsia; 27 of 64 (42.2%) SBAs knew to prepare pregnant women with severe pre-eclampsia for childbirth within 24 h, and 34 of 64 (53.1%) SBAs knew antihypertensive medication as essential action for management of severe pre-eclampsia (Table 3).

SBAs asked if women had recently or were currently experiencing severe headache or blurred vision in only 9 of 45 (20%) ANC consultations, 7 of 24 (29.2%) women observed during childbirth and 5 of 30 (16.7%) women observed in the postnatal ward. In 3 of 45 (6.7%) ANC and 6 of 30 (20%) PNC consultations, SBAs counseled women on the need for care seeking if experiencing convulsions or loss of consciousness. Likewise, they provided counseling on the need for care seeking if experiencing severe headache with blurred vision in 3 of 45 (6.7%) ANC consultations and 6 of 30 (20%) PNC consultations (Table 4).

Of 64 SBAs interviewed at private facilities, 45 (70.3%) reported that their supervisors treat them with respect and 47 (73.4%) mentioned a need for more knowledge and training. Security was not a concern among most SBAs interviewed; only 10 of 64 (15.6%) respondents stated good security in the workplace as an important factor in enabling environment for quality care service provision.

## Discussion

Our study shows numerous gaps in knowledge and practices required for early detection and management of PE/E at both public and private health facilities. Although most facilities had supplies and medicines required for early detection and management of PE/E, gaps were identified in availability of supplies, medicines and equipment in various health facilities.

All SBAs should know the essential actions in management of severe PE/E, which are administration of MgSO4, antihypertensive drugs, and timely childbirth [18]. Although most SBAs across all public and private health facilities correctly diagnosed PE/E based on case descriptions, almost half of them, however, did not recognize antihypertensive drug administration and timely delivery as essential actions. In contrast, the 2010 Afghanistan EmONC assessment findings showed that the majority of SBAs could describe  essential management of PE/E in case scenarios. In addition, trained SBAs had higher knowledge than untrained SBAs, and the majority of them had received EmONC training [9]. These findings demonstrate that SBA knowledge in this assessment might be lower than in the 2010 study. SBAs’ knowledge should be improved through evidence-based capacity-building approaches such as low-dose, high-frequency (LDHF) training that proved effective in Ghana [19].

The majority of maternal deaths due to PE/E could be averted with early detection and timely delivery – before reaching severe PE/E and eclampsia [20]. Our study found that not all women had their BP and proteinuria checked when needed, or danger signs assessed. Studies have shown that gaps between knowledge and clinical performance exist in part because of low motivation and health system constraints and training and supervision without a focus on effectiveness are unlikely to result in the improvements needed to ensure quality of care [21]. To change SBAs’ behavior, it may be important to focus on cognition, attitudes, and motivation of health care providers to improve quality of care [22].

Studies of PE/E case management in Nigeria and Pakistan identified similar gaps in quality of care. In Nigeria, use of MgSO4 was inconsistent in relation to standards [23]. In Pakistan, the majority of hospitals used MgSO4 as the preferred anticonvulsant to manage eclampsia, but there were large variations in dosages and in most cases doses were not in line with international guidelines [24]. In many settings, even when MgSO4 is available and providers know what drug to give, they struggle with its correct use.

Recent evidence supports multiple repetitive learning sessions that provide opportunities to practice skills and mechanisms for fostering interaction along with reinforcement of key messages as components of the learning support to improve quality of care [25].

In 2010, diazepam was used in almost half of the hospitals for management of PE/E [9]. Our findings, six years later, showed that the majority of SBAs recognized MgSO4 as the preferred anticonvulsant. This is consistent with findings from a study in Northern Afghanistan [26]. The positive shift from use of diazepam to MgSO4 over a period of time might be the result of a focused quality improvement program, which stimulated the translation of policies into practice in health facilities as well as distributing job aids and improving compliance with the national PE/E protocols [27].

Knowledge gaps demonstrated by more than half of SBAs in public health facilities and even more in private health facilities underscored the need for further investments in healthcare provider capacity building within quality improvement efforts.

Strategies found to be cost-effective in other settings include a combination of classroom learning and practice with simulators during and between repetitive sessions of peer-led practice and feedback. Follow-up via targeted Short Message Service (SMS) messaging and support by mentors will sustain the input [19, 28]. Recent evidence shows that a strategy for performance improvement of health care providers is more effective when training is combined with supervision and group based problem solving rather than training in isolation [29].

Policymakers and health managers need to review existing effective capacity-building approaches and develop a short-and long-term strategy for SBAs performance improvement across public and private health facilities. Ensuring good birth outcomes depend upon processes of care being performed correctly and consistently and complying with evidence based standards needs additional attention including monitoring progress [30].

Unavailability of essential medicines increases the risk of maternal and perinatal mortality and morbidity. The main reason for such shortages in Afghanistan could be supply chain management problems. Sharma et al. (2017) suggest that strengthening existing national logistics management systems to ensure adequate forecasting, supply, and availability of essential drugs and commodities through the establishment of web-based logistics management information systems could be a solution [31].

Many factors influence SBAs’ performance on quality intrapartum and postnatal care, such as access to training and supervision, workload, salaries and living conditions, and access to well-equipped and well-organized health facilities and transport [32]. In addition to lack of training opportunities, we found that lack of respectful supervision was a challenge for more than half of SBAs across public and private health facilities. This finding is consistent with study results in other low-resource settings [31].

Security is a key factor affecting SBAs’ performance in health facilities in Afghanistan. The security situation was stressed as an important factor affecting both the work environment and travel between home and the health facility in public health facilities. Since most private hospitals are located in the capitals of the provinces, there are fewer security concerns.

Although this study was not designed to provide a representative picture of private sector health facilities or directly compare readiness and quality of care in public versus private health facilities, a snapshot suggests that quality of care in early detection and management of PE/E might be similar.

### Limitations

This assessment had several limitations. Data collection at low- and high-volume facilities was not conducted concurrently, so it might not be possible to generalize influences of seasonal conditions and insecurity on health services. The data collected on health services provided in the three months preceding the assessment is based on facility management or health care worker verbal reports and not documentation of services provided or triangulation of multiple data sources. Finally, because the study was designed to assess the quality of many aspects of MNH care, some aspects of PE/E readiness, detection and case management practices, such as proteinuria and antihypertensive dosage for PE/E management, were not captured.

Although the data presented on private health facility readiness and service quality is not representative of all private facilities in Afghanistan and cannot be compared with nationally representative data from public health facilities, the anecdotal data presented may challenge assumptions that there are substantive differences in the quality of care provided at public and private sector facilities. There is a need for more rigorous and generalizable data on quality care provision at private facilities.

Despite these limitations, this study provides important evidence on gaps in quality of early detection and management of PE/E at public health facilities and some insight into the quality of care at private health facilities in Afghanistan.

## Conclusions

While the unavailability of essential medicines at some health facilities increases the risk of maternal and perinatal mortality and morbidity in both public and private health facilities, the major challenge in early detection and management of PE/E across all levels of public and private health facilities in Afghanistan is to close the gaps in SBA knowledge and practice.

Appropriate and effective solutions are available to make a paradigm shift in how SBAs are supported at all health facility levels to deliver lifesaving services to mothers and newborns. Policymakers and health partners should review existing SBA capacity-building approaches and adopt evidence-based and cost-effective repetitive training approaches. Strengthening the supply management system at national and health facility levels through a web-based logistic information system is required to ensure that all facilities have essential medicines and supplies in place.

## Abbreviations

ANC: Antenatal care
BEmONC: Basic emergency obstetric and newborn care
BHC: Basic health center
BP: Blood pressure
CHC: Comprehensive health center
DH: District hospital
EmONC: Emergency obstetric and newborn care
FHH: Family health house
LDHF: Low-dose, high-frequency
MCPC: Managing Complications in Pregnancy and Childbirth
MgSO4: Magnesium sulfate
MNH: Maternal and newborn health
MoPH: Ministry of public health
PE/E: Pre-eclampsia/eclampsia
PH: Provincial hospital
PNC: Postnatal care
RH: Regional hospital
SBA: Skilled birth attendant
SH: Specialized hospital
SHC: Sub-health center
WHO: World Health Organization
